# Low reduction of prostate volume is a significant predictor of prostate cancer at subsequent biopsy in patients with dutasteride: A retrospective study

**DOI:** 10.1111/and.13810

**Published:** 2020-08-20

**Authors:** Daisuke Obinata, Shugo Suzuki, Yataro Yamanaka, Tsuyoshi Yoshizawa, Junichi Mochida, Kenya Yamaguchi, Satoru Takahashi

**Affiliations:** ^1^ Department of Urology Nihon University School of Medicine Tokyo Japan

**Keywords:** dutasteride, prostate cancer, prostate volume, PSA

## Abstract

Appropriate decision of prostate biopsy in men with 5α‐reductase inhibitor (5AR inhibitor) is still unclear to avoid unnecessary biopsy. We retrospectively investigated patients with initial PSA 4.0 ng/ml or more and underwent subsequent prostate biopsy following dutasteride treatment. From September 2009 to August 2018, 399 cases of benign prostate hyperplasia (BPH) were treated with dutasteride in our department. Of the total, 36 cases with elevated pre‐treatment PSA (4.0 ng/ml or more) and underwent subsequent prostate biopsy were included into this study. We evaluated PSA kinetics and changing prostate volumes (PV), and detection of prostate cancer. Overall, average PSA reduced by half at 6 months from dosing. Pre‐treatment biopsy was performed in 17 of 36 cases, and all were diagnosed as having no malignancy. After treatment, prostate cancer was detected in 15 cases by subsequent biopsy. Fourteen of 15 cases were clinically significant cancer (Gleason score 7 or more). Logistic regression analysis detected a nominal association between prostate cancer detection and three variants, PSAD, PV reduction (1–Before/After PV) and abnormal MRI findings. In addition to abnormal MRI findings and pre‐treatment of high PSAD, the case with low reduction of PV after treatment should consider performing prostate biopsy.

## 1 INTRODUCTION

Prostate cancer is 6th leading cause of death from cancer amongst males in Japan, and the number of cases is increasing (Hori et al., 2015). In addition, the incidence of prostate cancer in Japan has already been estimated as the fourth of the most frequent cancers in 2018 (Hori et al., 2015). The measurement of prostate‐specific antigen (PSA) is the most used chemical biomarker for the detection of prostate cancer, and prostate biopsy is known to the most accurate diagnostic technique to detect prostate cancer (Serefoglu et al., 2013). However, PSA is also elevated in some cases with benign hyperplasia or prostatitis. The detection rate by biopsy varies according to PSA level, and around 25% of patients met cancer with sole PSA in the range between 2 and 10 μg/L (Filella & Foj, 2016; Yii et al., 2019). Therefore, both approaches to reduce prostate cancer risk and avoid unnecessary prostate biopsy are reasonable and promising.

Dutasteride, one of the 5α‐reductase (5AR) inhibitors, is well known to reduce prostate cancer risk. 5AR inhibitors prevent the conversion from testosterone to 5‐α‐dihydrotestosterone (DHT) which induces prostate mitotic activity and potentially carcinogenesis. Unlike castration, 5AR inhibitors reduce DHT with reciprocal testosterone increase. Inhibition of 5AR results in decreased DHT and prostate volume, increased peak urinary flow rates and improvement in lower urinary symptoms scores (Miller & Tarter, 2007). Dutasteride blocks both type 1 and 2 5AR, and on the other hand, finasteride, another 5AR inhibitor, blocks only type 2 5AR (Frye, 2006).

There are several randomised trials to assess the efficacy of 5AR inhibitors for prostate cancer chemoprevention (Andriole et al., 2010; Thompson et al., 2003). More specifically, REDUCE trial showed that whilst 659 of 4,105 cases have prostate cancer in dutasteride group, 858 of 4,126 cases in placebo group (Andriole et al., 2010). According to a Cochrane systematic review about these trials, 5AR inhibitors showed 25% relative risk reduction compared to placebo (Wilt et al., 2010). Although this systematic review showed definitive evidence of prostate cancer risk reduction by 5AR inhibitors, it also suggested that appropriate PSA adjustments and indications for prostate biopsy in men on 5AR inhibitor are still unclear to avoid unnecessary biopsy (Wilt et al., 2010).

In this study, we retrospectively investigated patients with initial PSA above 4.0 ng/ml and underwent subsequent prostate biopsy following dutasteride treatment.

## 2 METHODS

**Figure 1:**
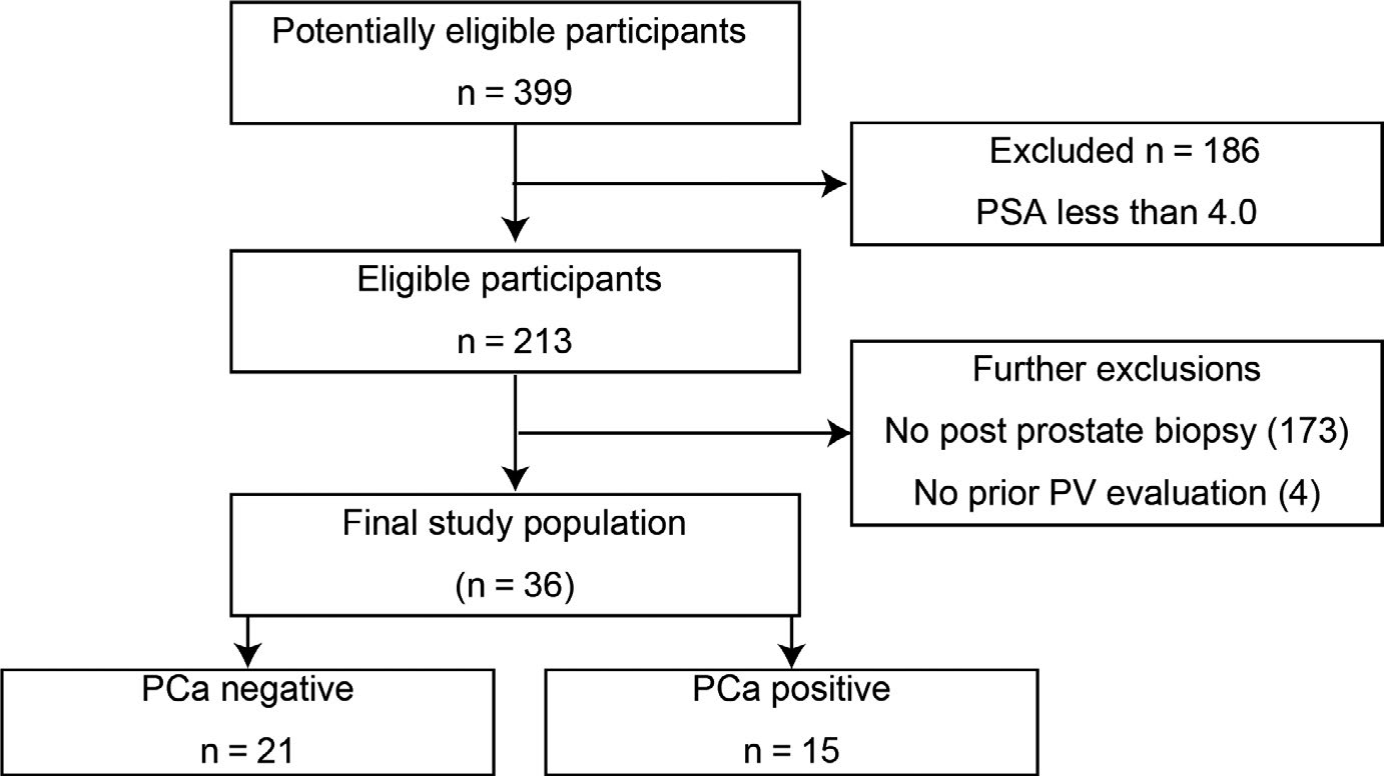
The flowchart of included and excluded BPH cases treatment with dutasteride for the study

This study was approved by the Institutional Review Board and Research Ethics Committee of Nihon University School of Medicine. In our institution, consecutive 399 cases with benign prostate hyperplasia (BPH) underwent treatment with dutasteride from September 2009 to August 2018. All cases received dutasteride for the first time. Amongst them, 213 cases showed initial PSA above 4.0 ng/ml before dutasteride treatment. We retrospectively investigated 40 of 213 cases who underwent subsequent prostate biopsy, and 4 cases were excluded due to missing prior prostate volume (PV) data (Figure 1). Subsequent prostate biopsy performed using transrectal ultrasonography (TRUS) for the cases which met any of the indications as follows: (a) PSA reduction (1–Before/After PSA) smaller than 50% at six months after dutasteride, (b) a suspicious lesion identified by DRE and (c) a suspicious imaging defined by ultrasound or MRI. Once a prostate cancer was detected, we excluded the case and discontinued dutasteride. We evaluated pre‐treatment PSA, PSA at 6 months from treatment, pre‐treatment PV, PV at subsequent biopsy, dosing period and detection rate of prostate cancer. Statistical analyses were performed using GraphPad Prism for Mac version 6 (GraphPad Software, Inc., La Jolla, CA, USA) and JMP® version 9 (SAS Institute Japan, Inc., Tokyo, Japan). Continuous data are presented as mean ± *SD*. Student's *t* test was used to seek differences in continuous data across dichotomous categories. A chi‐square analysis and the Fisher exact test were used for categorical variables. Logistic nominal regression analysis was performed to study area under curve (AUC) and 95% confidence intervals (CIs) for associations between variables and prostate cancer detection. The area under the receiver operating characteristic (ROC) curve was used to find the cut‐off value of each variable for prostate cancer detection. *p*‐values < .05 were considered statistically significant. This study was approved by the Institutional Review Board and Research Ethics Committee of Nihon University School of Medicine.

## 3 RESULTS

In the total 36 cases, the mean treatment period was 44.68 ± 30.23 months. Amongst them, 17 cases underwent TRUS‐guided prostate biopsy before treatment (pre‐treatment biopsy). All of them had no malignancy histologically. Table 1 demonstrates baseline characteristics in bulk of all (*n* = 36). The mean age of the study population was 69.69 ± 7.85 years old. Overall, PSA reduced by half 6 months after dutasteride (11.88 ± 8.75 to 6.25 ± 3.90 ng/ml). After treatment, prostate cancer was detected in 15 of 36 cases with subsequent biopsy. Fourteen of 15 cases were clinically significant cancer (Gleason Score 7 or more). There were no significant differences in PSA and PV before and after treatment, number of cases with MRI prior biopsy, positive biopsy and the distribution of Gleason Scores between them (Table 1).

**Table 1:** Patient characteristics

	Total cases (*n* = 36)	No pre‐biopsy (*n* = 19)	Pre‐biopsy (*n* = 17)	*p* Value
Age	69.69 (7.85)	70.05 (9.57)	69.29 (5.60)	.77
Duration (m)	44.68 (30.23)	36.50 (25.71)	53.35 (32.93)	.09
PSA (ng/mL)				
Before	11.88 (8.75)	13.77 (10.99)	9.77 (4.77)	.17
6M After	6.25 (3.90)	6.25 (4.45)	6.26 (3.31)	.99
PV (mL)				
Before	52.36 (23.41)	55.47 (25.25)	48.88 (21.39)	.4
After	40.33 (22.52)	40.84 (23.37)	39.76 (22.24)	.88
MRI before biopsy	33	18	15	.47
Prostate cancer	15	8	7	.95
Gleason Scores				
3 + 3	1	0	1	.29
3 + 4	4	2	2	
4 + 3	2	2	0	
4 + 4	5	2	3	
4 + 5	1	1	0	
5 + 4	1	0	1	
5 + 5	1	1	0	

**Figure 2:**
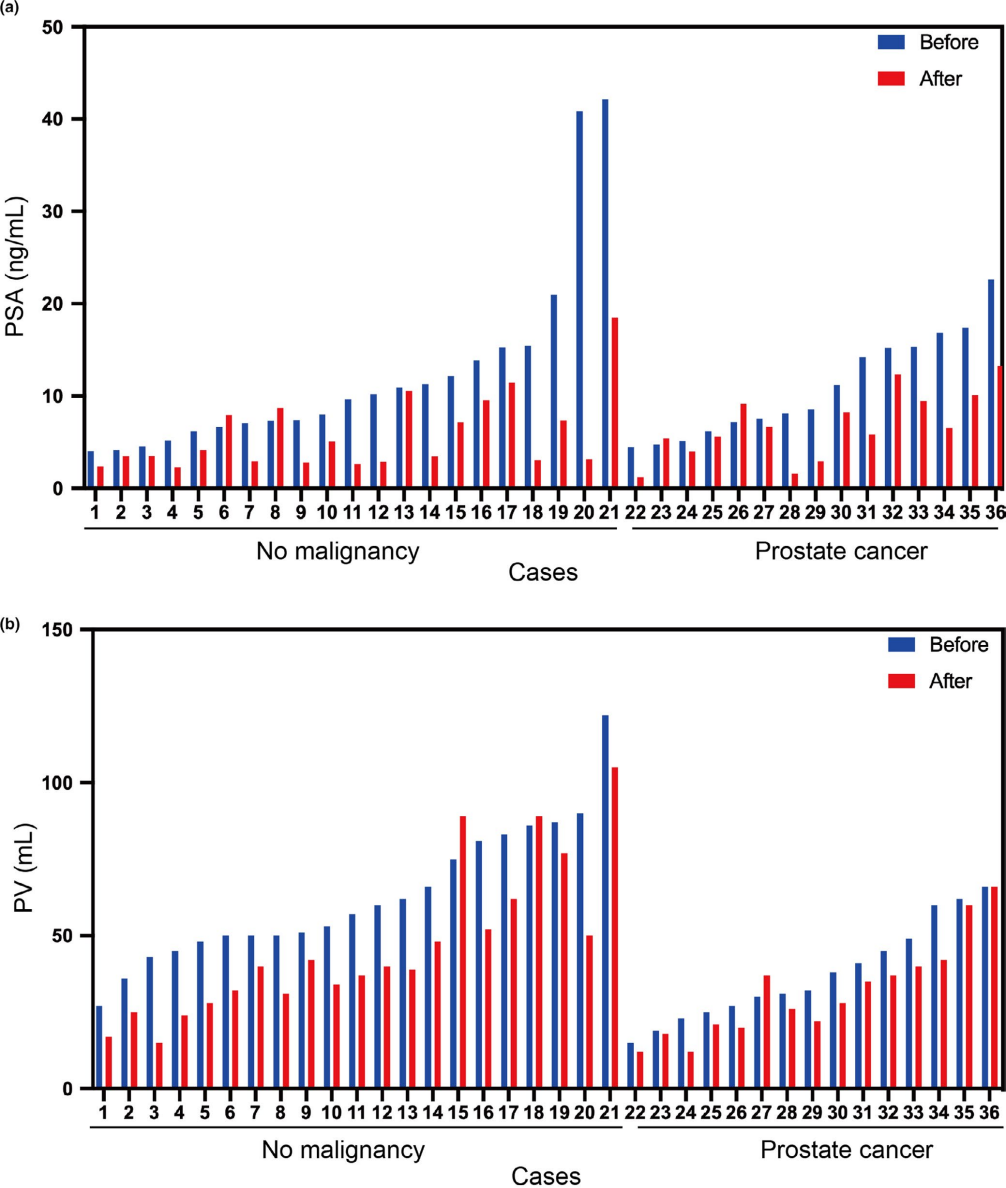
Representative matched cases by values of before and after PSA (a) and Prostate volume (b)

The differences in clinical parameters between cases with confirmed prostate cancer by subsequent biopsy or cases without were evaluated. There were no differences in PSA before and after treatment, and reduction. On the other hand, prostate cancer cases showed significant increased number of cases with abnormal MRI findings, lower mean pre‐treatment PV and reduction, and significant higher PSA density (PSAD) than no malignancy cases (Figure 2a and b, Table 2, 9 versus 3 cases, *p* = .0037, 37.53 ± 15.97 versus 62.95 ± 22.31 ml, *p* = .006, 0.16 ± 0.16 versus 0.29 ± 0.18, *p* = .041, 0.31 ± 0.16 versus 0.20 ± 0.12, *p* = .020).

**Table 2:** Comparison between no malignancy and prostate cancer

	No malignancy (*n* = 21)	Prostate cancer (*n* = 15)	*p* Value
Age	67.80 (6.64)	72.33 (8.85)	.08
Duration (m)	52.09 (34.44)	33.57 (18.55)	.07
MRI abnormal finding	3	9	.0037
PSA (ng/mL)			
Before	12.53 (10.54)	10.98 (5.58)	.6
6M After	5.85 (4.12)	6.82 (3.62)	.47
Reduction rate	0.42 (0.30)	0.33 (0.31)	.4
PV (mL)			
Before	62.95 (22.31)	37.53 (15.97)	.0006
After	46.47 (24.79)	31.73 (15.97)	.051
Reduction	0.29 (0.18)	0.16 (0.16)	.041
PSAD before treatment	0.20 (0.12)	0.31 (0.16)	.02

**Figure 3:**
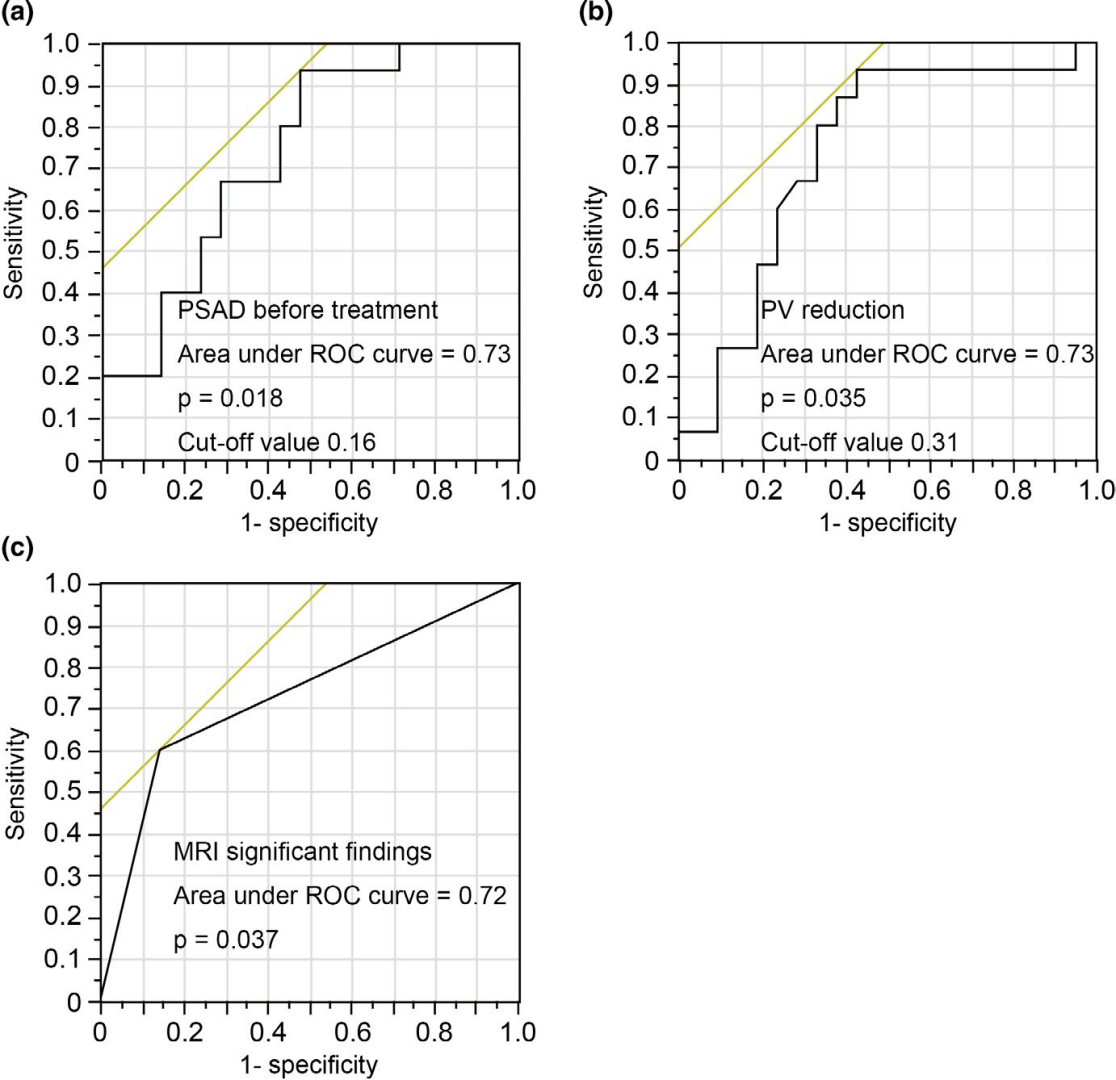
ROC curves for PSAD (a), PV reduction (b) and MRI significant findings (c) to detect prostate cancer

To find appropriate prediction factors for prostate cancer detection, we performed logistic nominal regression analysis for abnormal MRI findings, PSAD and PV reduction. The AUC of each parameters were abnormal MRI findings; 0.72 (95% CI: 0.34, 1.97, *p* = .037), PSAD; 0.73, (95% CI: 0.90, 11.67, *p* = .018), and PV reduction; and 0.72 (95% CI: −9.43, −0.27, *p* = .035; Figure 3a, b and c). Based on this AUC, the sensitivity, specificity, positive and negative predictive values (PPV and NPV) at abnormal MRI findings, PSAD and PV reduction were evaluated. Significant MRI findings predict prostate cancer with sensitivity 60%, specificity 86%, PPV 75% and NPV 75%. From the Youden index, the optimum cut‐off of PSAD and PV reduction was 0.16 with sensitivity 93%, specificity 53%, PPV 58%, NPV 91%, and 0.31, sensitivity 93%, specificity 58%, PPV 60% and NPV 92% (Table 3).

**Table 3:** Results of ROC analysis

Variable	Sensitivity	Specificity	PPV	NPV	AUC (95%CI)	*p* Value
MRI findings	60	86	75	75	0.72 (0.34, 1.97)	.0037
PSAD (>0.16)	93	53	58	91	0.76 (0.90, 11.67)	.018
PVR^a^ (<0.31)	93	58	60	92	0.73 (−9.43, −0.27)	.035

^a^ PV reduction.

## 4 DISCUSSION

This study investigated the importance of PSA kinetics and prostate volume reduction in the dutasteride treatment for the prediction of prostate cancer. Our study identified cases with significant MRI findings, PSAD 1.6 or higher, and low PV reduction less than 0.31 as a risk of prostate cancer. Routine screening for prostate cancer with PSA is well known to detect prostate cancer at early stage where the standard treatment is curable (Lowe, Gilbert, & Kahane, 2003). Although TRUS‐guided prostate biopsy is important to confirm histopathology, it sometimes causes minor complications as pain, bleeding, urinary tract infections and urinary retention (Volanis, Neal, Warren, & Gnanapragasam, 2015). Furthermore, PSA screening with prostate biopsy might associate with over‐detection and over‐treatment of indolent cancers (Ilic, Neuberger, Djulbegovic, & Dahm, 2013). Recent systematic review demonstrated that PSA screening has a small reduction of disease‐specific mortality but not improve overall mortality (Ilic et al., 2018). Following these results about PSA screening and prostate biopsy, the US Preventive Services Task Force (USPSTF) stated that they do not recommend PSA‐based screening for prostate cancer in men of 70 years or older (D recommendation), whilst under 70 years as C recommendation in 2018 (Force et al., 2018). Therefore, it would be important to prevent indolent cancer and to clarify selection criteria of biopsy to reduce the number of unnecessary biopsies.

Consistent with recent studies that abnormal MRI findings significantly related to prostate cancer (Futterer et al., 2015; Le et al., 2015), our data also showed number of cases with abnormal MRI findings was significantly increased in prostate cancer.

In addition to MRI findings, we focused on efficacy of dutasteride‐ for PSA‐related parameters, PV and prostate cancer detection by subsequent biopsy. According to the subgroup analysis of Asian men in the REDUCE study, 5 of 54 (9.3%) on dutasteride had prostate cancer by second biopsy, on the other hand, and 11 of 56 (19.6%) in placebo cases (Akaza et al., 2011; Andriole et al., 2010). Our data showed higher detection rate (41.6%) than Asian cohort in REDUCE study. Interestingly, although the mean observation period of these two study are similar (44.68 months in present study versus 48 months in REDUCE study), pre‐treatment PSA and the prevalence of high grade prostate cancer were higher in our study (PSA: 11.88 versus 6.0, high grade: 8 of 15 (53%) versus 0 of 5 (0%)) (Akaza et al., 2011). Similar to the Prostate Cancer Prevention Trial (PCPT) which is a clinical trial using finasteride (Thompson et al., 2003), present study included worse phenotypes of prostate cancer. In PCPT, Gleason Scores between 7 and 10 were found in 37.0% of the tumours in the finasteride group, and in 22.2% of the placebo group. Unlike PCPT, all cases in REDUCE trial underwent pre‐treatment prostate biopsy at least 6 months before treatment. This study included both cases with/ without pre‐treatment biopsy, and we found no difference in the prevalence of worse phenotypes between them. One of the reason for this is the difference in inclusion criteria for PSA, which is defined as 2.5–10.0 ng/ml (50–60 years) or 3.0–10.0 ng/ml (over 60 years) in REDUCE study (Akaza et al., 2011). In addition, we underwent the biopsy to the cases with highly suspected prostate cancer using traditional criterion. These data indicate that novel predictors combined with traditional standards would be able to increase the detection rate over 40%.

Since 5AR inhibitors reduce approximate 50% of PSA from baseline at 6 months after the treatments (Roehrborn et al., 2002) and the cases in our study have a wide range of PSA baseline, we focused on PSA kinetics and prostate volume reduction rather than PSA level at 6 months. To our knowledge, this is the first study to find the significant association between PSAD and PV reduction by dutasteride and prostate cancer detection. Since previous review indicated that dutasteride might reduce indolent prostate cancer and make it easy to detect significant cancer because of benign tissue shrinkage (Nacusi & Tindall, 2011), high PSAD means abnormal PSA production by malignant cells and low PV reduction means dutasteride is unable to decrease malignant tissue.

PSA reduction was not significant predictor for prostate cancer detection in this study because this has already included as a subsequent biopsy criteria. Furthermore, one study from Italy reported about the relationship between PSA reduction by dutasteride and prostate cancer (Sciarra et al., 2017). They showed that PSA reduction after 6 months of treatment was not a significant indicator, but persistent prostatic inflammation found by prostate biopsy is the important predictor (Sciarra et al., 2017).

Several limitations of this study include its retrospective fashion, small number of subsequent biopsy cases and sole racial factor (only Japanese cases were evaluated).

## 5 CONCLUSION

Our study suggests that high PSAD and low PV reduction, addition to MRI findings, are significant prediction factors for prostate cancer detection and should consider prostate biopsy. Further studies will be needed to verify these findings.

## AUTHORS' CONTRIBUTIONS

DO and SS equally contributed. DO, SS, KY and ST contributed to conception. DO, YY and KY contributed to design of the work. SS, JM, YY and TY contributed to the acquisition and analysis. DO, SS, KY and ST contributed to interpretation of data. DO wrote the first draft of the manuscript. All authors agreed with manuscript results and conclusions. All authors reviewed and approved of the final manuscript.

## Data Availability

The data that support the findings of this study are available from the corresponding author [KY], upon reasonable request.
